# The heritable landscape of near-infrared and Raman spectroscopic measurements to improve lipid content in Atlantic salmon fillets

**DOI:** 10.1186/s12711-021-00605-6

**Published:** 2021-02-05

**Authors:** Gareth F. Difford, Siri S. Horn, Katinka R. Dankel, Bente Ruyter, Binyam S. Dagnachew, Borghild Hillestad, Anna K. Sonesson, Nils K. Afseth

**Affiliations:** 1 Nofima, Norwegian Institute for Food, Fisheries and Aquaculture Research, NO-1433 Ås, Norway; 2 Benchmark Genetics Norway AS, Sandviksboder 3A, NO-5035 Bergen, Norway

## Abstract

**Background:**

Product quality and production efficiency of Atlantic salmon are, to a large extent, influenced by the deposition and depletion of lipid reserves. Fillet lipid content is a heritable trait and is unfavourably correlated with growth, thus genetic management of fillet lipid content is needed for sustained genetic progress in these two traits. The laboratory-based reference method for recording fillet lipid content is highly accurate and precise but, at the same time, expensive, time-consuming, and destructive. Here, we test the use of rapid and cheaper vibrational spectroscopy methods, namely near-infrared (NIR) and Raman spectroscopy both as individual phenotypes and phenotypic predictors of lipid content in Atlantic salmon.

**Results:**

Remarkably, 827 of the 1500 individual Raman variables (i.e. Raman shifts) of the Raman spectrum were significantly heritable (heritability (h^2^) ranging from 0.15 to 0.65). Similarly, 407 of the 2696 NIR spectral landscape variables (i.e. wavelengths) were significantly heritable (h^2^ = 0.27–0.40). Both Raman and NIR spectral landscapes had significantly heritable regions, which are also informative in spectroscopic predictions of lipid content. Partial least square predicted lipid content using Raman and NIR spectra were highly concordant and highly genetically correlated with the lipid content values (${r}_{\text{g}}$ = 0.91–0.98) obtained with the reference method using Lin’s concordance correlation coefficient (CCC = 0.63–0.90), and were significantly heritable (${h}^{2}$ = 0.52–0.67).

**Conclusions:**

Both NIR and Raman spectral landscapes show substantial additive genetic variation and are highly genetically correlated with the reference method. These findings lay down the foundation for rapid spectroscopic measurement of lipid content in salmonid breeding programmes.

## Background

Product quality and production efficiency of Atlantic salmon are, to a large extent, influenced by the deposition and depletion of lipid reserves. Atlantic salmon store the majority of their ingested energy as intra-muscular lipid, which influences grading, processing, nutritional value, texture, colour, flavour and thereby consumer acceptance and market value of the fish [1–3]. Excess lipid is stored around the viscera and constitutes a high-cost slaughter waste [4]. The average lipid content in salmon fillet increases linearly with increasing dietary levels of crude fat up to a certain point after which residual reserves are stored around the viscera [5]. In spite of this, Atlantic salmon reared under the same conditions and on the same diet have markedly different fillet lipid contents, which often vary by more than 20 percentage points (Table 1).

**Table 1:** Summary of heritability estimates for lipid content in Atlantic salmon and various measurement, sampling and available population descriptions in the literature

Population	N	Harvest weight (kg)	Dietary fat (%)	Muscle	Method	Lipid % mean (SD)	Range (%)	${h}^{2}$	Study
Akvaforsk 1984	667	6.4 ± 1.9	17–18%	NQC	CT	16.7 ± 2.7	11.0–22.0^b^	0.30 ± 0.09^a^	[16]
Akvaforsk 1985	604	6.9 ± 1.8	17–18%	NQC	CT	14.5 ± 2.4	9.8–19.2^b^	0.30 ± 0.09^a^	[16]
ASBDP	472	6.7 ± 1.5	NR	RSGC	NIR	15.9 ± 2.0	6.1–21.1	0.19	[7]
MOWI 2002	136	3.9 ± 0.1^c^	NR	NQC	NIR	9.6 ± 2.5	2.8–14.7	0.21 ± 0.03^a^	[48]
MOWI 2003	19	5.3 ± 0.3	NR	NQC	NIR	14.7 ± 3.2	7.6–20.0	0.21 ± 0.03^a^	[48]
MOWI 2008	50	4.4 ± 1.4	27–37%	NQC	CE	12.6 ± 1.6	9.5–15.6^b^	NA	[49]
MOWI 2008 × WILD	50	3.13 ± 0.8	27–37%	NQC	CE	12.1 ± 1.1	10.0–14.2^b^	NA	[49]
WILD	50	2.0 ± 0.5	27–37%	NQC	CE	11.7 ± 1.3	9.2–14.3^b^	NA	[49]
AquaGen	2428	NR	31–35%	Fillet	NIR	NR	NR	0.28	[50]
Landcatch 2004	1682	1.98 ± 0.8	NR	RS8	DFM	13.5 ± 4.3	5.5–21.8^b^	0.28 ± 0.05	[8]
Landcatch 2002	1697	2.57 ± 0.6	NR	RS8	DFM	12.2 ± 5.6	1.3–23.2	0.18 ± 0.03	[51]
SalmoBreed 2001	2634	3.16 ± 0.7	NR	NQC	NR	12.6 ± 1.4	5.4–17.1	NR	[52]
SalmoBreed 2014	668	3.6 ± 0.9	36%	NQC	CE	19.1 ± 3.4	5.5–27.7	0.46 ± 0.10	[13]

*N*  number of fish, *NQC*  Norwegian quality cut, *RSGC*  right side behind gill cover, *RS8*  8 random samples over entire body, *CT* computerised X-ray tomography, *NIR* near infrared, *CE* chemical extraction, *DFM* Distell fat meter, *NR* not reported, *NA* not applicable. ${h}^{2}$heritability

^a^Indicate h^2^ estimates from joint year classes

^b^Indicates range estimated from mean ± 1.96*standard deviation

^c^Indicates gutted weight

Fillet lipid content is a heritable trait that is unfavourably correlated with growth [6–8]. Since growth is the most highly weighted trait in Atlantic salmon breeding programmes [9], selection indices and economic weighting of fillet lipid content are needed to achieve a genetic gain in growth without undesirable correlated responses in fillet lipid content [10], which can have knock-on effects on product quality and production efficiency. Conversely, evidence from other salmonid species indicates that selection for increased growth with restricted or controlled fillet lipid content improves feed efficiency, which is also a trait of high economic value [11].

In order to control for fillet lipid content in breeding programmes, it is necessary to measure this trait on thousands of related individuals under commercial conditions. Direct measurement of lipid traits is challenging because most measurement methods are carried out *post-mortem*, which requires sacrificing the fish, and in some cases the fillet. The gold standard method for measuring lipid content in salmon fillets is the chemical extraction of lipids using ethyl acetate, which was first described by [12]. As the gold standard or reference method, this method is the most accurate and precise measurement from which all other methods are benchmarked, and it is regarded as providing the ‘True’ value. However, it is time-consuming, costly, and is classified as a destructive method because both the fish and the fillet are sacrificed in the process. To the best of our knowledge, direct genetic evaluations using the reference method for measurement of fillet lipid have only recently been reported for Atlantic salmon [13].

Numerous alternative methods for recording fillet lipid content are under development, each with a unique set of advantages and disadvantages, and scope of applications. These can be broadly divided into: (1) non-destructive methods on live fish such as near-infrared (NIR) spectroscopy [14], Distell fat meter (Distell, UK), and low-field nuclear magnetic resonance (NMR) [15], and (2) fillet non-destructive methods such as computerised X-ray tomography (CT) [16, 17], PhotoFish, a commercialised camera system with controlled lighting (AKVA group, Norway), and online NIR spectroscopy [18].

Both NIR and Raman spectroscopy are techniques that fall within the family of vibrational spectroscopy methods. These techniques are rapid, require little to no sample preparation and can be automated, which make them well suited to the high-throughput phenotyping needs of modern breeding programmes and management operations. However, these two techniques rely on different mechanisms for inducing molecular vibrations and, thus, they provide different chemical data (see [19] for a comprehensive review). Whereas Raman spectroscopy typically provides higher resolution chemical information than NIR spectroscopy, NIR spectroscopy is the most routinely used method to record lipid contents in salmon. Among other reasons, this is related to the lack of appropriate sampling tools for collecting representative Raman spectra of heterogeneous samples [19]. NIR and Raman spectroscopy rely on calibration equations that are generated with the reference method that is measured on ~ 50 to 100 salmon fillets. Then, the accuracy of the calibration equations is tested through prediction on validation sets. Previous studies on lipid content estimation using NIR spectroscopy suggest that estimation errors range from 0.5 to 1.0% [20]. For Raman spectroscopy, very few studies have been reported in the literature. Wold et al. [21] used a spherical lens probe for efficient Raman sampling, and obtained good results for the prediction of fat contents in ground salmon with a cross-validation correlation (R_CV_) of 0.95 and a root mean squared error of cross-validation (RMSECV) of 1.6%. These values suggest that lipid contents in Atlantic salmon fillet that are obtained with the reference method and those that are predicted by using Raman or NIR vibrational spectroscopy should be phenotypically nearly equivalent. Remarkably, the existence or lack of genetic equivalence between the ‘True’ lipid content and that obtained with alternative methods has not been established, although fillet lipid content is included in the selection programmes of multiple breeding populations (Table 1). Furthermore, the phenotypic agreement or concordance between NIR and Raman predicted lipid phenotypes and lipid contents recorded with the reference method has not been reported for large-scale genetic cohorts. Since NIR and Raman predicted phenotypes are linear combinations of thousands of measured spectra which can themselves be considered as phenotypes, it follows that the individual spectra maybe be heritable and genetically correlated with true lipid content. Thus, developing an optimal strategy for using NIR and Raman spectroscopy measurements in genetic evaluations requires estimation of heritabilities at individual NIR wavelengths and Raman shifts, and of their phenotypic and genetic relationships with the reference value for fillet lipid contents. Therefore, the objectives of our study were to (1) estimate genetic parameters for NIR and Raman spectroscopy measurements in Atlantic salmon fillet, and (2) estimate genetic parameters between the reference fillet lipid content and predicted lipid content using NIR and Raman phenotypes and their components.

## Methods

### Fish population

As part of commercial breeding operations, SalmoBreed AS (today named Benchmark Genetics Norway AS) created 194 full-sibling families using 92 sires and 194 dams for the 2014 year-class. Details on animal husbandry and feeding are in [13, 22]. All fish were reared under the same conditions. Briefly, families were reared separately until a mean weight of 113.1 g, and the fish were transferred to sea cages after smoltification. After being fed on a high fish oil content broodstock feed (LifePhase Vitalis, Skretting AS, Norway), at a mean weight of 3605 g and approximately 12 months at sea, 668 fish were fasted for 13 to 14 days and then harvested and filleted. Fish were screened for sexual maturation and those that were sexually mature were excluded from further analysis (31 males), and external deformities and individual filleting were also recorded. The pedigree included 1685 animals and included four generations of information on direct ancestors of the fish included in our study.

### Trait recording

Four lipid traits were recorded on each individual and are described in chronological order as follows: (1) reference method lipid content (Lipid_True_), (2) commercial NIR system lipid content (Lipid_FieldNIR_), (3) laboratory-based NIR system (Lipid_NIR_), and (4) laboratory-based Raman system (Lipid_Raman_) lipid contents. At slaughter, the sex of each individual was determined by visual inspection of the gonads and recorded. NIR absorbance spectra were recorded immediately after slaughter on fillets (non-destructively) using a commercial NIR imaging scanner (QVision500, Tomra Sorting Solutions, Leuven, Belgium), which immediately gives the predicted lipid content of the fillet in the field (Lipid_FieldNIR_) [23]. The NIR absorbance spectra and the prediction equation used to predict lipid content are proprietary and not available to researchers. Muscle samples were taken from the Norwegian Quality Cut (NQC), frozen and stored at − 20 °C. Total lipids were extracted from homogenized NQC muscle samples from each fish, and lipid content was determined in g of lipid per 100 g of muscle tissue calculated according to the method described by [12] (Lipid_True_). Then, homogenized muscle samples were recorded for laboratory-based Raman and NIR spectroscopy analysis as described below.

Raman spectra were obtained using a Kaiser RamanRXN2™ Multi-channel Raman analyzer (Kaiser Optical Systems, Inc., Ann Arbor, MI, USA) with a spectral resolution of 5 cm^−1^. The spectrometer was equipped with a 785 nm laser and PhAT probe with a laser spot size diameter of 6 mm. The spectra were recorded with a laser power set to 400 mW within the range of 300 to 1890 cm^−1^ with 0.3-cm^−1^ intervals. Each spectrum was an average of four 15 s accumulations. All homogenized muscle samples were randomized, and three replicate measurements were obtained for each sample. The instrument was controlled using the iC Raman version X software (Mettler Toledo, Greifensee, Switzerland). For pre-processing, extended multiplicative signal correction (EMSC) with a sixth order polynomial extension was used [24]. In short, the spectra were trimmed within the 500 to 1800 cm^−1^ range, and EMSC was performed on all replicate spectra. The mean spectrum of all the replicate spectra was subjected to polynomial baseline correction (fourth order) and used as a reference in EMSC. The average Raman spectrum for each sample was subsequently calculated and used for further analysis.

Diffuse reflectance near-infrared spectra of homogenized salmon samples were obtained using the FOSS NIRSystems XDS Rapid Content™ Analyzer (FOSS Analytical A/S, Hillerød, Denmark). NIR spectra were obtained in reflectance mode with 32 scans per spectrum. Homogenised samples were randomised and measured in triplicates, with the average spectrum used for further analysis. An internal ceramic standard was used as a reference. The spectral range was from 400 to 2500 nm with 0.5 nm increments. In order to follow Beer’s law, the NIR spectra were transformed from reflectance (R) units into absorbance (A = log_10_(1/R)) and standard normal variate (SNV) pre-processed units. For subsequent analyses, the spectral range from 1150 to 2500 nm was used since it covers the main information related to lipids in the NIR spectrum [25].

### Statistical analysis

Predictions of lipid content phenotypes from Raman and NIR spectra were performed independently by partial least squares regression (PLSR) using the PLS package in R [26]. After merging all the data, 523 samples were available with data from all analytical sources used in the study (i.e. phenotypic and spectroscopic data). On average, in the remaining dataset each sire had five offspring. The Raman and NIR data were divided into a random calibration set of 60 individuals in order to keep the calibration size consistent with commercial applications and a validation set of 463 individuals. In order to avoid over- or underfitting in the final PLSR model, the optimal number of PLSR components for the final PLSR model was chosen based on the lowest root mean squared error of cross-validation (RMSECV) using 10 random sets of ‘leave one out’ cross-validation within the calibration set. For the Raman data, the optimal number of components was 4, and these were used to predict lipid content in the full validation set, resulting in a RMSECV of 0.97% for the calibration set and 1.34% for the validation set. The coefficient of determination (R^2^) for the calibration and validation sets were 0.92, and 0.81, respectively. For the NIR spectra, the optimal number of components was 7, and these were used to predict lipid content in the validation set, resulting in a RMSECV of 1.04% and 1.32% in the calibration and validation sets, respectively. The R^2^ for the calibration and validation sets were 0.93 and 0.84, respectively. Raman (Lipid_Raman_) and NIR (Lipid_NIR_) predicted lipid contents were used as phenotypes in the genetic analyses presented below.

The overall phenotypic agreement between predicted fillet lipid contents and true lipid content was assessed by Lin’s concordance correlation coefficient (CCC) [27].

Variance components were estimated by applying univariate animal models using average information criterion restricted maximum likelihood models in DMU version 6 [28]:

1 $${\mathbf{y}} = {\mathbf{X}{\text{b}}}+ {\mathbf{Z}}{\text{a}} + {\mathbf{e}}$$

where $\bf{y}$ is the vector of phenotypes for individual $i=\text{1,2},3\dots n$; i.e. 1300 phenotypes for Raman shift values, 2300 phenotypes for NIR absorbance values, and the four lipid content phenotypes (Lipid_True_, Lipid_FieldNIR_, Lipid_NIR_, and Lipid_Raman_). The incidence matrix $\mathbf{X}$ links phenotype $\mathbf{y}$ to the fixed effect of sex (two levels, male and female). Potential fixed effects of filleter (7 levels) and spinal deformity (2 levels) were found to be non-significant (α < 0.05) and thus were not included in subsequent analyses. **a** is the vector of genetic effects assumed to be normally distributed $\mathbf{a}\sim \text{N }(0, \mathbf{A}{\upsigma }_{\text{a}}^{2}$), where $\mathbf{A}$ is the pedigree derived numerator relationship matrix and ${\upsigma }_{\text{a}}^{2}$ the additive genetic variance.$\mathbf{Z}$ is the incidence matrix that links observations to genetic effects, and $\mathbf{e}$ is the vector of random residuals and follows a normal distribution $\mathbf{e}\sim \text{N }(0,\mathbf{I}{\upsigma }_{\text{e}}^{2})$, where $\mathbf{I}$ is the identity matrix and ${\upsigma }_{\text{e}}^{2}$ the residual variance. Heritability (${h}^{2}$) estimates were calculated as the ratio of additive genetic variance to total phenotypic variance ${\upsigma }_{\text{a}}^{2}/{(\upsigma }_{\text{a}}^{2}+{\upsigma }_{\text{e}}^{2})$ and their standard errors by using a Taylor series approximation. Statistical *p* values were computed for each heritability estimate following $t$ distributions and then adjusted for false discovery rate (FDR) using the Benjamini–Hochberg procedure [29].

Bivariate animal models were run pairwise between the four lipid content traits and followed the same form as Eq. (1) assuming the following additive genetic and residual covariance structures:

2 $${var} \left[ {\begin{array}{*{20}{c}} {{\mathbf{a}_j}} \\ {{\mathbf{a}_{True}}} \end{array}} \right] = \;\left[ {\begin{array}{*{20}{c}} {\mathbf{A}\sigma _{aj}^2}&{\mathbf{A}{\sigma _{ajaTrue}}} \\ {\mathbf{A}{\sigma _{aTrueaj}}}&{\mathbf{A}\sigma _{aTrue}^2} \end{array}} \right]$$

3 $${var} \left( \mathbf{e} \right)\; = \;R\; = \left[ {\begin{array}{*{20}{c}} {\mathbf{I}\sigma _{ej}^2}&{\mathbf{I}{\sigma _{ejeTrue}}} \\ {\mathbf{I}{\sigma _{eTrueej}}}&{\mathbf{I}\sigma _{{e_{True}}}^2} \end{array}} \right],$$

where $j$ indicates the alternative lipid content traits (Lipid_NIRField_, Lipid_NIR_ and Lipid_Raman_) and $\text{True}$ indicates the trait Lipid_True_ obtained by the reference method. Genetic and phenotypic correlations were estimated as the covariance divided by the square root of the product of two variances for the additive genetic and phenotypic (co)variances, respectively. The genetic correlation of individual Raman shifts and NIR wavelengths with Lipid_True_, was estimated as the correlation between their respective EBV from Eq. (1) corrected by their respective reliabilities using the Calo method [30, 31].

## Results

### Descriptive statistics

The descriptive statistics for the lipid content traits are in Table 2. The means of the alternative lipid traits ranged from 18.9 to 21.0% and were not significantly statistically different from Lipid_True_ 19.07% (P > 0.05). All lipid phenotypes were substantially heritable (${h}^{2}$ = 0.51–0.67). The phenotypic correlations deviated from 1 (0.80–0.92) and the concordance correlation coefficients ranged from 0.63 to 0.92, and all genetic correlations were higher than 0.90 (ranging from 0.91 to 0.98) (Table 2).

**Table 2:** Descriptive statistics and genetic and phenotypic parameters for lipid content traits recorded on 523 Atlantic salmon

Traits	Descriptive statistics				Correlations with Lipid_True_		
	Mean ± SD	CV %	Min—Max	${h}^{2}$± SE	Phenotypic	Genetic	CCC
Lipid_True_	19.07 ± 3.16	17	5.45–26.9	0.51 ± 0.11			
Lipid_FieldNIR_	21.0 ± 2.42	12	10.4–28.3	0.67 ± 0.12	0.80 ± 0.03	0.91 ± 0.04	0.63
Lipid_NIR_	18.9 ± 2.90	14	5.6–26.8	0.60 ± 0.10	0.80 ± 0.03	0.93 ± 0.03	0.68
Lipid_Raman_	19.0 ± 2.84	15	0.6–24.0	0.52 ± 0.12	0.92 ± 0.02	0.98 ± 0.01	0.92

*SD* standard deviation, *CV*  coefficient of variation, *h*^*2*^ heritability, *CCC*  Lin’s concordance correlation coefficient

### Heritability estimates

**Fig. 1:**
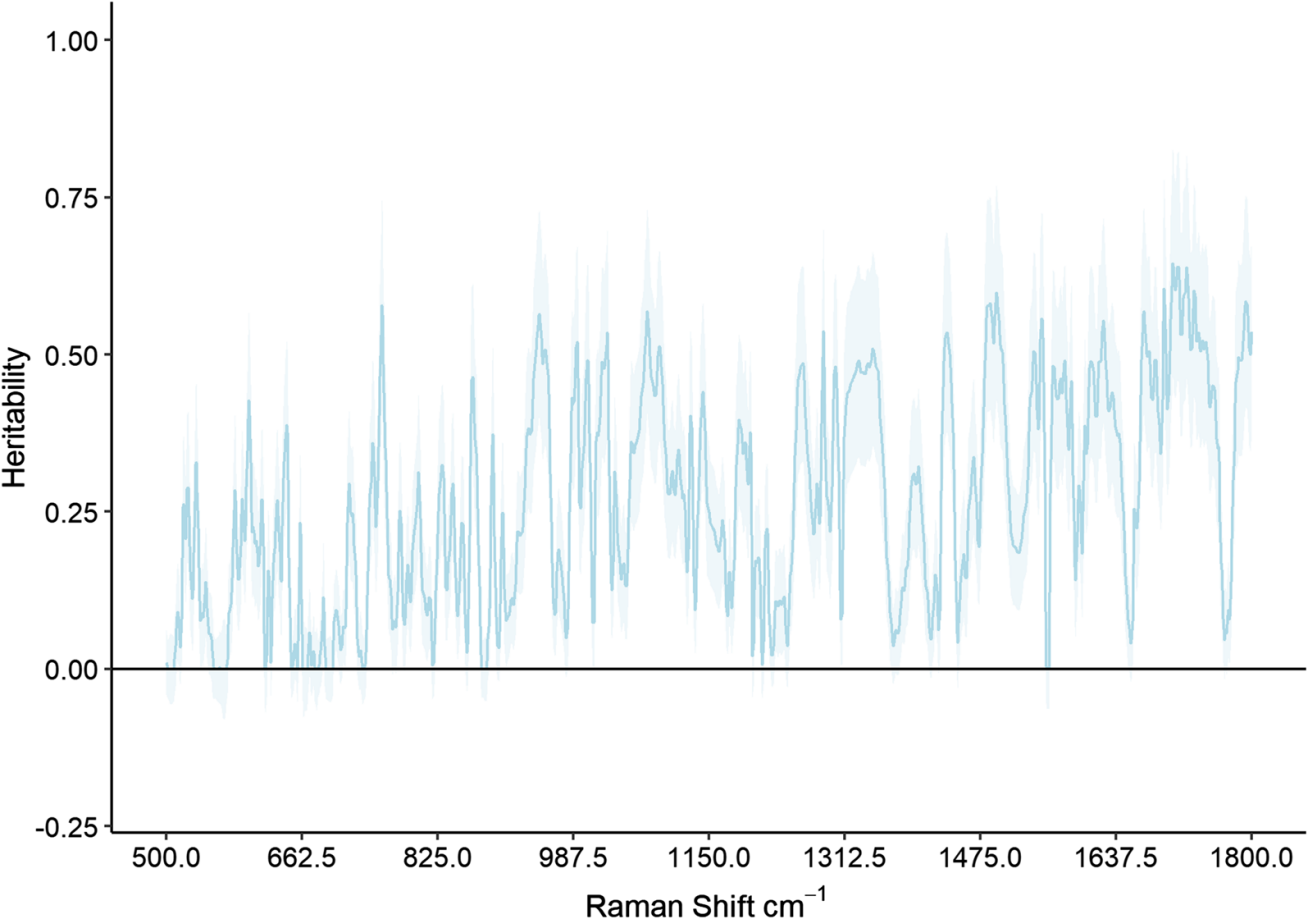
Heritability (${h}^{2}$) blue solid line with standard errors (light blue shading) for 1300 individual wavenumbers from the Raman shift region from 500 to 1800 cm^−1^ in Atlantic salmon fillets. Solid black line denotes the origin (0)

The heritability estimates for individual Raman shifts from 500 to 1800 cm^−1^ are presented in Fig. 1, and the highest ${h}^{2}$ reached 0.65. In total, 827 Raman shifts were significantly heritable at α = 0.05 after correction for a false discovery rate (FDR) of 10%. Previous studies on the prediction of lipid content of salmon fillet and control mock samples with known varying amounts of water and lipid involved eight spectral bands [25, 32, 33]. Several heritability estimates were significant within these spectral bands, i.e. for the: (1) C–C stretch 1064–1068 cm^−1^ with ${h}^{2}$ = 0.39; (2) C–C stretch 1076–1081 cm^−1^ with ${h}^{2}$ = 0.52; (3) C–C stretch 1126–1130 cm^−1^ with ${h}^{2}$ = 0.35; (4) symmetric = C-H rocking 1263–1267 cm^−1^ with ${h}^{2}$ = 0.44; (5) CH_2_ twist 1300–1305 cm^−1^ with ${h}^{2}$ = 0.43; (6) CH_2_ scissoring 1440–1445 cm^−1^ with ${h}^{2}$ = 0.38; (7) *cis* C = C stretch and amide I 1656–1660 cm^−1^ with ${h}^{2}$ = 0.25; and (8) C = O stretch 1744–1749 cm^−1^ with ${h}^{2}$ = 0.39 (See Additional file 1: Table S1).

**Fig. 2:**
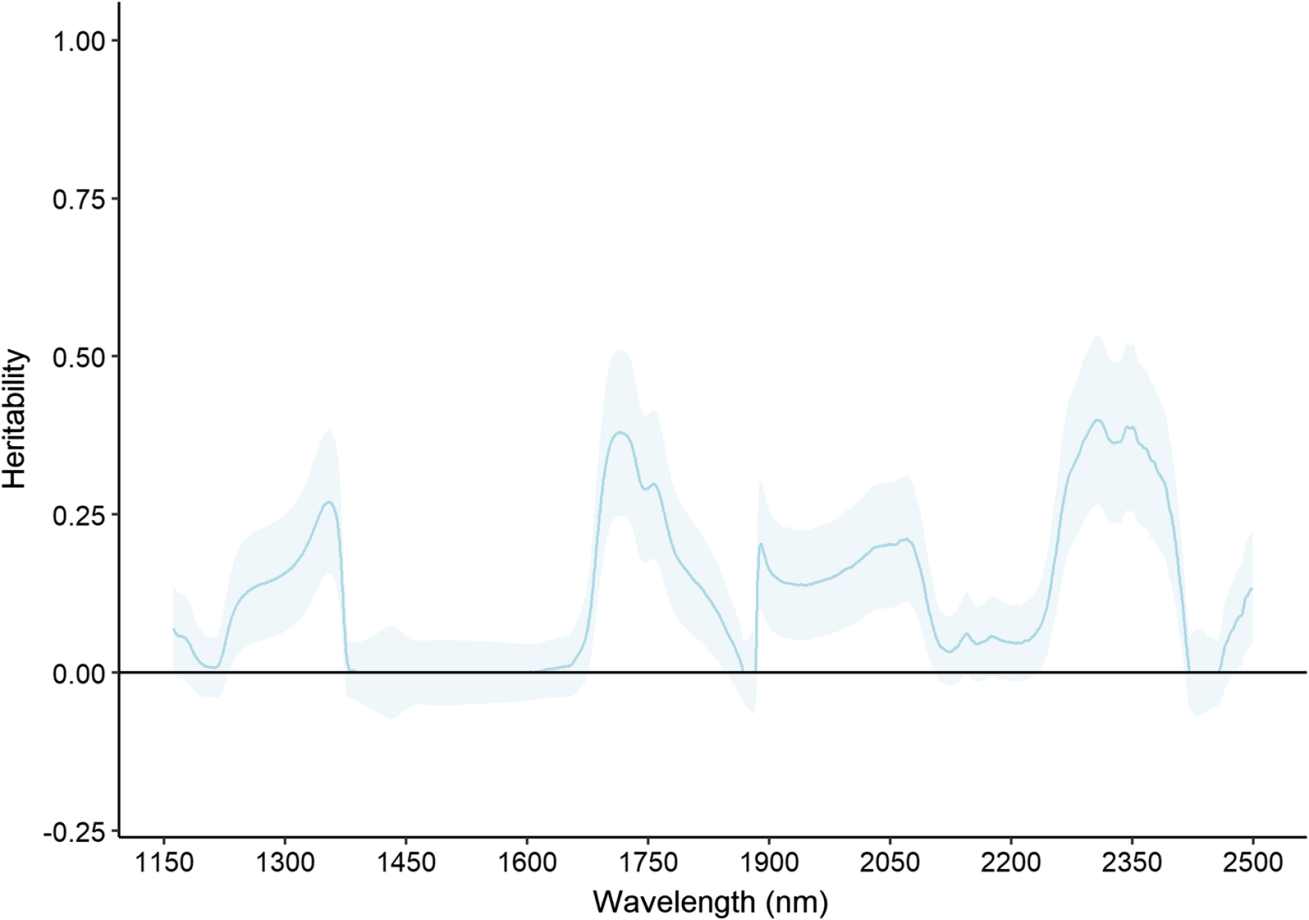
Heritability (h^2^) blue solid line with standard errors (light blue shading) for 2700 individual wavenumbers from the NIR wavelength region from 1150 to 2500 cm^−1^ in Atlantic salmon fillets. Solid black line denotes the origin (0)

The heritability estimates for individual NIR wavelengths between 1150 and 2500 nm are presented in Fig. 2. The highest ${h}^{2}$ was moderate at 0.40. In total, 407 individual wavelengths were significantly heritable at α = 0.05 after correction for a FDR of 10%. Based on the well characterised associations between NIR spectral bands and different chemical bonds, and on control mock samples of varying lipid and water content, it was possible to screen NIR regions that are known to be relevant for predicting lipid content [25]. Several spectral bands associated with chemical bonds that are informative for lipid content were also significantly heritable; for example, the first asymmetric and symmetric C-H stretch overtones at 1715 nm (${h}^{2}$ = 0.38) and 1760 nm (${h}^{2}$ = 0.29), and the combination bands of the C-H stretch and deformation at 2310 nm (${h}^{2}$ = 0.40) and 2350 nm (${h}^{2}$ = 0.39). However, no significant genetic variation was observed for the combination bands for *cis* unsaturation in carbon chains at 2140 nm and 2180 nm. The first O–H overtone at 1450 nm, the O–H stretch 1930 nm and the deformation at 1190 nm associated with water content were not significantly heritable (${h}^{2}$ = 0.00–0.13) (see Additional file 2: Table S2, Additional file 3: Table S3 for Raman shift EBV and Additional file 4: Table S4 for NIR EBV).

### Correlations with lipid content

**Fig. 3:**
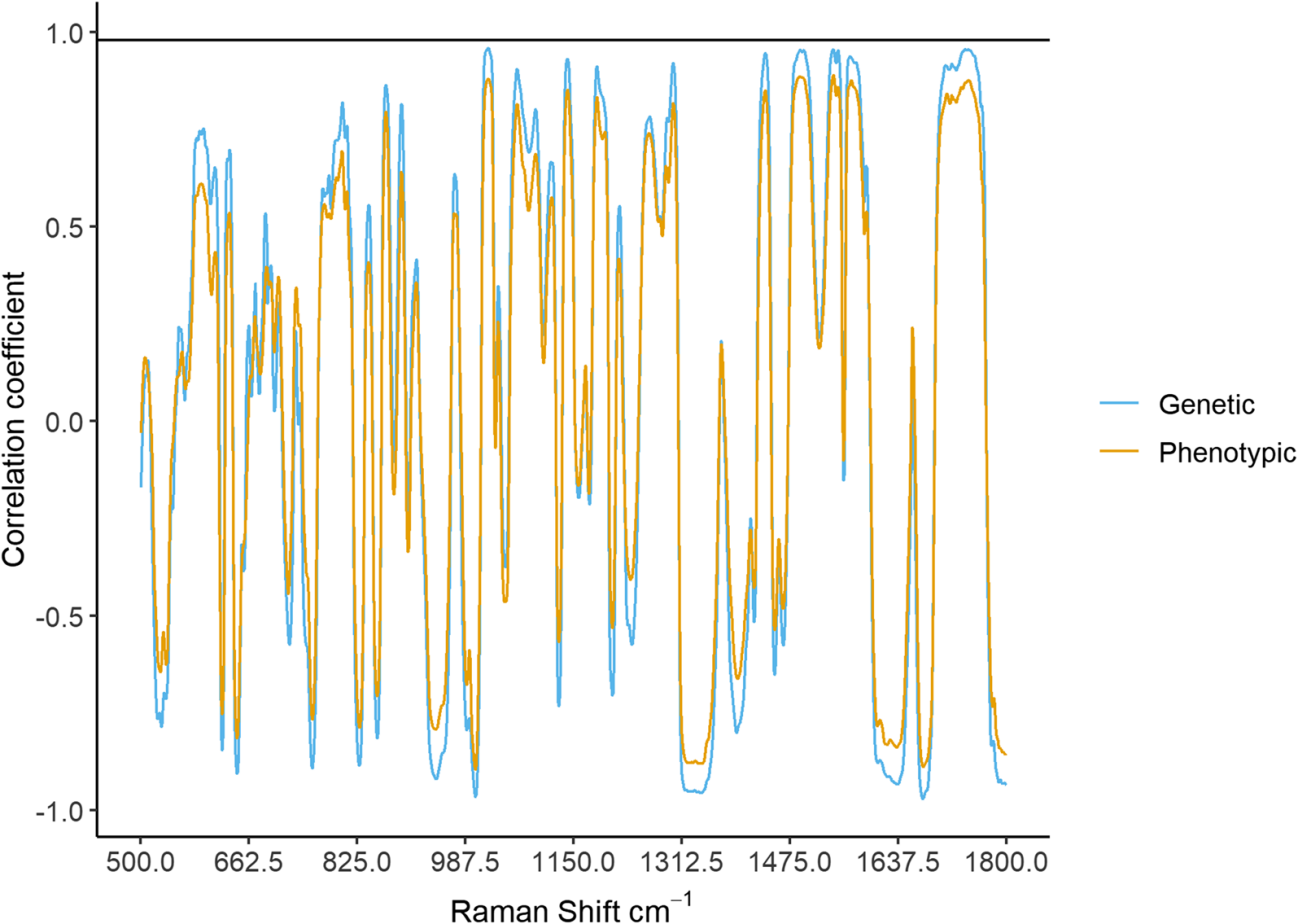
Genetic (light blue) and phenotypic (yellow) correlations between each individual Raman shift and lipid content. The genetic correlation between Raman predicted lipid content and true lipid content indicated by the solid black line

The genetic and phenotypic correlations of each Raman shift with Lipid_True_ content are shown on Fig. 3. The patterns of phenotypic and genetic correlations were consistent in sign and magnitude across the Raman shift range. The estimated genetic correlations of Lipid_True_ content with several spectral bands associated with various chemical bonds in lipids were strong and positive; for example, with the C–C stretch at 1064–1068 cm^−1^ (${r}_{\text{g}}$ = 0.90), the C–C stretch at 1076–1081 cm^−1^ (${r}_{\text{g}}$ = 0.73), the symmetric = C-H rocking at 1263–1267 cm^−1^ (${r}_{\text{g}}$ =0.77), the CH_2_ twist at 1300–1305 cm^−1^ (${r}_{\text{g}}$ = 0.85), the CH_2_ scissoring at 1440–1445 cm^−1^ (${r}_{\text{g}}$ = 0.80) and the C = O stretch at 1744–1749 cm^−1^ (${r}_{\text{g}}$ = 0.95). The C–C stretch at 1126–1130 cm^−1^ had a strong negative genetic correlation with Lipid_True_ content (${r}_{\text{g}}$ = − 0.69) and the *cis* C = C stretch and amide I at 1656–1660 cm^−1^ had weak non-significant correlations with LipidTrue content (${r}_{\text{g}}$ = −0.06 and ${r}_{\text{p}}$ = 0.09) (see Additional file 5: Table S5 for Raman shift correlations).

**Fig. 4:**
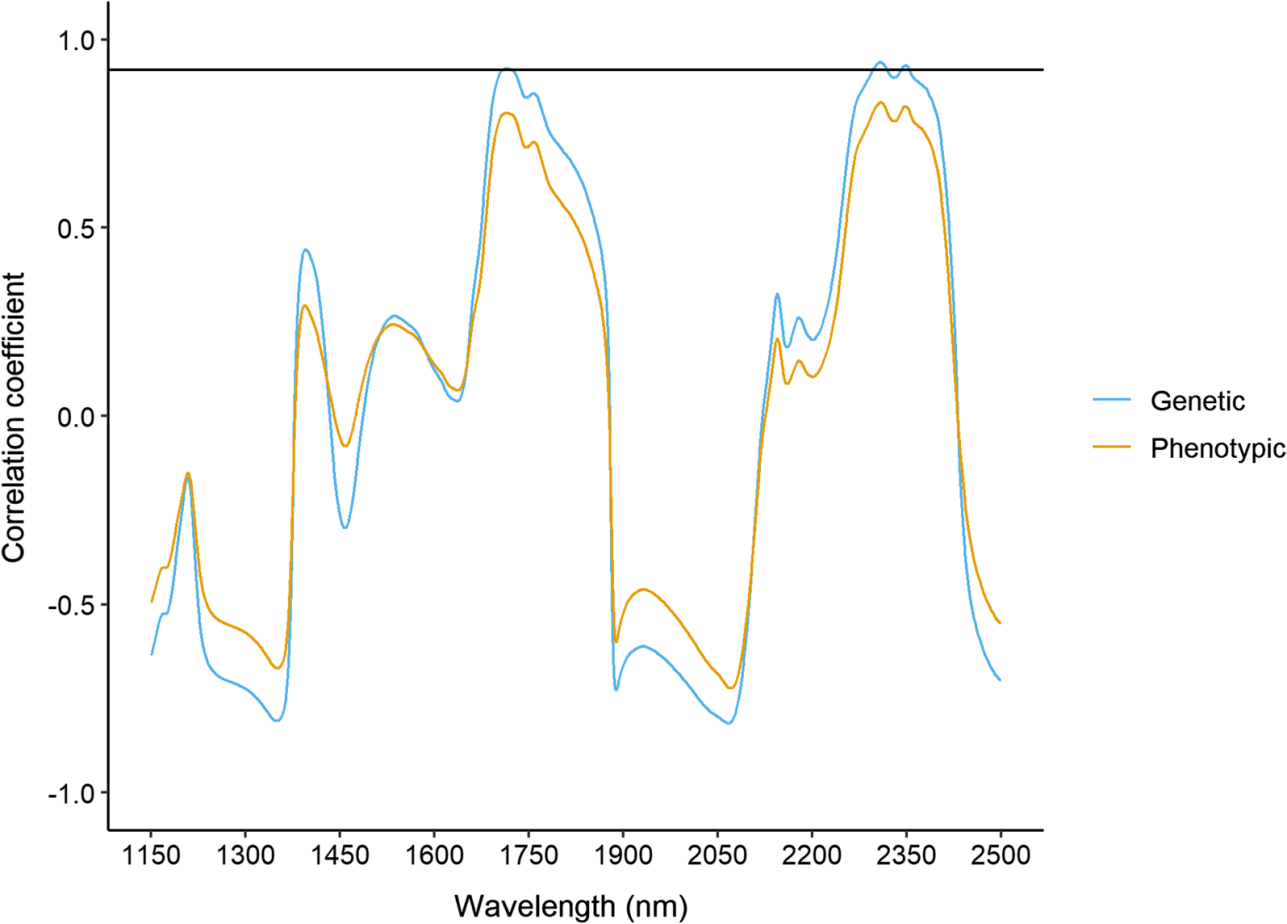
Genetic (light blue) and phenotypic (yellow) correlations between each NIR wavelength and lipid content. The genetic correlation between NIR predicted lipid content and true lipid content indicated by the solid black line

The genetic and phenotypic correlations of each NIR wavelength with Lipid_True_ content and Lipid_NIR_ are presented in Fig. 4. The pattern of phenotypic and genetic correlations was consistent in sign and magnitude across the NIR wavelength range. The estimated genetic correlations of Lipid_True_ content and Lipid_NIR_ with several spectral bands associated with various chemical bonds in lipids were strong and positive; for example, with the first asymmetric and symmetric C-H stretch overtones at 1715 nm (${r}_{\text{g}}$ = 0.92) and 1760 nm (${r}_{\text{g}}$ = 0.86) and the combination bands of C-H stretch and deformations at 2310 nm (${r}_{\text{g}}$ =0.94) and 2350 nm (${r}_{\text{g}}$ = 0.90). The spectral regions associated with water content had genetic and phenotypic correlations between Lipid_True_ content and Lipid_NIR_ close to zero or moderately negative in sign, such as the O–H overtone at 1450 nm (${r}_{\text{g}}$ = − 0.26 and ${r}_{\text{p}}$ = − 0.05), the O–H stretch at 1930 nm (${r}_{\text{g}}$ = − 0.61 and ${r}_{\text{p}}$ = −0.46) and the O–H deformation at 1190 nm (${r}_{\text{g}}$ = −0.37 and r_p_ = − 0.29). The correlations for the *cis* unsaturation combinations at 2140 nm and 2180 nm were weak for both the genetic (0.27–0.46) and phenotypic (0.15–0.32) correlations (see Additional file 6: Table S6 for NIR wavelength correlations).

## Discussion

### The heritable landscape of Raman and NIR vibrational spectra

To our knowledge, this is the first study that directly estimates the heritability of Raman and NIR vibrational spectra in muscle tissue. Remarkably, both Raman and NIR spectral landscapes contained bands for which the estimated heritability was quite high, i.e. bands with known associations with chemical bonds and functional groups. For instance, 827 individual Raman shifts had significant heritabilites ${h}^{2}$ that ranged from 0.15 to 0.65, and the majority of these are in regions of fundamental stretching and deformation models that belong to covalent carbon to carbon, oxygen and hydrogen bonds present in lipids [25]. Similarly, 407 NIR wavelengths had significant heritabilities that ranged from 0.27 to 0.40 and were in regions associated with oxygen to hydrogen, carbon to hydrogen and carbon to carbon bonds [32].

The heritability estimates reported here are comparable or higher than those for mid-Fourier transform infrared spectra (FTIR), which are already used for routine genetic evaluations in the milk of dairy goats (${h}^{2}$ = 0.14–0.28) [34] and of multiple dairy cow populations (${h}^{2}$ = 0.01–0.63) [35–37]. Since Raman and NIR spectroscopic measurements are generally regarded as complementary techniques, and are well adapted to rapid and online measurements, finding significantly heritable regions from both methods holds promise for routine genetic evaluations in Atlantic salmon and other aquaculture species and warrants further investigation in muscle tissues of terrestrial livestock.

### Phenotyping lipid content

Accurate phenotypes are a prerequisite for accurate breeding values. Using alternative methods for phenotyping lipid content in Atlantic salmon has been an active field of research since the publication of the first pioneering works using NIR and computerised X-ray tomography in salmonids [16, 39, 40]. However, phenotyping lipid content has value to producers beyond breeding practices, e.g., as response variables to treatments in experiments and management interventions as well as grading and pricing of whole fish or fillets under commercial conditions. Different scopes of application will have different requirements for accuracy, precision and correlation values, and in method agreement studies, it is common practice to estimate agreement indices which combine all three sources of (dis)agreement into a single statistic for comparisons [40]. We assessed overall agreement by Lin’s concordance correlation coefficient (CCC), which penalises the Pearson’s correlation coefficient for differences in variances (precision) and means (accuracy) between two methods [41]. We found high CCC for Lipid_Raman_ (CCC = 0.92), followed by Lipid_NIR_ (CCC = 0.68) and Lipid_FieldNIR_ with (CCC = 0.62) which were both moderately high. In applications for which rapid and non-destructive measurements on fillets are needed, a producer might be willing to forgo some accuracy and precision to achieve this in practice, and the agreement between alternative methods and the reference method enables this. For instance, the two methods with the highest CCC were Lipid_Raman_ and Lipid_NIR,_ but they were conducted on homogenised samples in the laboratory. Whereas CCC was lower for Lipid_FieldNIR_, it is currently the only rapid and non-destructive commercially available solution. For small-scale experiments, for which accuracy and precision are required, it is still recommended to use the reference method or the reference method in combination with NIR or Raman spectroscopy. Currently, promising new instrumental developments are being reported for rapid measurement of lipid content on live fish with both Raman and NIR spectroscopy, which may result in alternative commercial solutions in the future [14, 42]. For NIR, this is related to the so-called interactance principle, an optical configuration that allows photons that have travelled deeper into biological tissues to be collected [14]. For Raman, a similar approach, termed spatially-offset Raman spectroscopy, is currently being explored [42].

From a genetic evaluation perspective, the genetic parameters such as estimates of heritability and genetic correlations between alternative methods and the reference method are needed to inform the choice of method. However, genetic correlations between true lipid content and NIR predicted lipid content have not been reported to date. The best predictor of a phenotype is not necessarily the best predictor of genetic merit for the ‘true’ trait [37]. If non-genetic factors or residual correlations are driving the phenotypic prediction, it is possible that selecting on a predicted phenotype could yield poor or even undesired responses to selection. This is best illustrated with the FTIR prediction of stearic acid (C18:0), which is a fatty acid in the milk of dairy cows with a coefficient of determination R^2^ of 0.80 that indicates that predicting this phenotype is feasible. However, the genetic correlation estimated between the reference method and C18:0 predicted with FTIR was highly negative − 0.86 [43]. If selection had been implemented based on the predicted C18:0 without knowledge of this negative genetic correlation, the response to selection in the true C18:0 would likely have been in the opposite direction than intended. Clearly, this highlights the value of validating the genetic correlations between true phenotype and predicted phenotype prior to genetic selection. The genetic correlations between alternative methods and the reference methods reported here were very high and close to 1 (r_g_ = 0.93–0.98) with high heritability estimates (h^2^ = 0.52–0.67), which indicates that almost all the genetic variation in Lipid_True_ is captured by the alternative methods. The threshold for genetic correlations between alternative and reference methods, which is called the “break-even correlation”, is traditionally set at 0.70–0.80 [45, 46]. Alternative methods with genetic correlations to the reference method below this threshold can be regarded as indicator traits and not genetically equivalent to the reference method, whereas methods that exceed this threshold can be used in direct genetic evaluations. The distinction is rather subtle, but it has implications for how traits are incorporated into the selection index as either the direct trait or an indicator trait, and it affects the genetic gain in the direct trait and the costs of phenotyping. In our study, the genetic correlations between the three alternative methods and the reference method (r_g_ = 0.93–0.98) are high and close to 1. As a further validation, we assessed the phenotypic and genetic correlations between Lipid_True_ and individual Raman shifts and NIR wavelengths. The phenotypic and genetic correlations for both methods were nearly equivalent in both sign and magnitude. Furthermore, some spectral regions associated with chemical bonds found in lipids in Atlantic salmon had genetic correlations with Lipid_True_ higher than 0.80. Based on these findings, it is reasonable to conclude that vibrational spectroscopic methods in the field or the laboratory are nearly equivalent to the reference method and can be used in genetic selection programmes for fillet lipid content, provided the calibration equations based on the reference methods are accurately estimated.

### Raman and NIR vibrational spectroscopy beyond selection for lipid content

Raman and NIR vibrational spectroscopy are able to detect relative amounts of numerous biochemical molecular bonds, and this offers possibilities for new phenotypes other than lipid content. It is already well established that NIR and Raman spectroscopy can be used to predict carotenoid content (astaxanthin and canthaxanthin) in the salmon fillet, which is responsible for the characteristic pink colour and is an important quality characteristic [14, 21]. Studies have even reported that the heritability of NIR-predicted canthaxanthin content is significant and ranged from 0.07 to 0.29 in Atlantic salmon fillets [13, 47]. Furthermore, Raman spectroscopy has been reported for the detection of aromatic amino acids content such as phenylalanine, tryptophan, histidine and tyrosine [47]. The most promising application is the partitioning of total lipid content into detailed fatty acids content. Health associated omega-3 fatty acids such as eicosapentaenoic and docosahexaenoic acids have been reported as significantly heritable in Atlantic salmon (${h}^{2}$ = 0.23 and 0.46, respectively) [13]. Prediction of the fatty acid composition of Atlantic salmon fillet using NIR and Raman spectroscopy is an active field of research [25]. Further research is needed to assess the phenotypic scope of Raman and NIR vibrational spectroscopy in modern high-throughput breeding programmes.

As the use of rapid online vibrational spectroscopy gains traction for phenotyping in modern breeding programmes, another avenue of research is to reconcile at which level (phenotypic or genetic) the vibrational spectra are dimensionally reduced and the partial least squares predictions made. This is because even the largest breeding programmes in the world cannot phenotype a sufficient number of individuals necessary to estimate variance components and EBV in multi-trait mixed models for thousands of individual NIR wavelengths or Raman shifts. For instance, in dairy cattle and goats, the vibrational spectra are reduced to latent variables at the phenotypic level and used in PLSR to predict the phenotypes of interest. Then, in a second step, the predicted phenotypes are analysed in univariate mixed models to estimate EBV, which is termed indirect prediction [34]. An alternative approach is to reduce the vibrational spectra to latent variables and estimate variance components and EBV on the latent variables directly in a multi-trait mixed model, termed direct prediction [34]. Direct prediction has been reported to increase the reliability of EBV and reduce error variance in some applications but indirect prediction is the most common model in routine genetic evaluations. Although further research is needed to evaluate the factors that govern optimal use of direct or indirect predictions, the heritability estimates obtained in our study for individual NIR and Raman spectral landscapes lay down the foundation for genetic evaluations using vibrational spectral in animal tissues.

## Conclusions

Specific Raman shifts and NIR wavelengths in the vibrational spectral landscapes recorded on Atlantic salmon fillets were significantly heritable (0.15–0.65). The concordance correlation coefficients (CCC) between Raman and NIR predicted fillet lipid contents and the values obtained with the reference method ranged from moderate (0.63) to high values (0.90), which validates the use of the former as phenotypes. Furthermore, the genetic correlations of Raman and NIR predicted lipid contents with the values obtained with the reference method were close to unity (${r}_{\text{g}}$ = 0.91–0.98) and thus can be used in genetic evaluations. Spectral regions in both Raman and NIR could be assigned to chemical bonds of known importance in lipids. Both Raman and NIR vibrational spectra hold promise for high-throughput phenotyping and genetic evaluation of lipid content in the muscle of Atlantic salmon.

## Supplementary Information

**Additional file 1: Table S1.** Estimated variance components for each individual Raman shift

**Additional file 2: Table S2.** Estimated variance components for each individual NIR wavelength.

**Additional file 3: Table S3.**Estimated breeding values (EBV) for each individual Raman shift.

**Additional file 4: Table S4.** Estimated breeding values (EBV) for each individual NIR wavelength,

**Additional file 5: Table S5.** Estimated phenotypic and genetic correlations between each individual Raman shift and the reference lipid value.

**Additional file 6: Table S6.** Estimated phenotypic and genetic correlations between each individual NIR wavelength and the reference lipid value.

## Data Availability

The datasets generated and analysed during the current study are contained within the additional files, the remaining commercially sensitive data are available on reasonable request from Benchmark Genetics Norway AS.
